# Host Dietary Nutrients Shape GH32-Mediated Microbial Responses to Prebiotic Fructans: A Randomized Trial

**DOI:** 10.3390/foods14234090

**Published:** 2025-11-28

**Authors:** Hideaki Takahashi, Tadashi Fujii, Chikako Yamada, Nobuhiro Kondo, Kento Kuramitsu, Kohei Funasaka, Eizaburo Ohno, Yoshiki Hirooka, Takumi Tochio, Kotoyo Fujiki

**Affiliations:** 1 Graduate School of Nutritional Sciences, Nagoya University of Arts and Sciences, Nisshin 470-0196, Japan; 2 Department of Gastroenterology and Hepatology, Fujita Health University, Toyoake 470-1192, Japan; 3 BIOSIS Lab. Co., Ltd., Toyoake 470-1192, Japan; 4 Department of Medical Research on Prebiotics and Probiotics, Fujita Health University, Toyoake 470-1192, Japan; 5 WELLNEO SUGAR Co., Ltd., Chuo-ku, Tokyo 103-8536, Japan; 6 Department of Applied Biosciences, Graduate School of Bioagricultural Sciences, Nagoya University, Nagoya 464-8601, Japan

**Keywords:** prebiotics, gut microbiota, *Bifidobacterium adolescentis*, *Bifidobacterium longum*, glycoside hydrolase family 32 (GH32)

## Abstract

Prebiotics, such as short- and long-chain fructans, beneficially modulate the microbiota; however, individual variability in response remains unclear. In this randomized, double-blind, placebo-controlled trial, 40 healthy adults received either a combined fructan supplement—1-Kestose (Kes) and inulin (Inu)—or a placebo (maltose + cornstarch) for 4 weeks. We investigated the fecal microbiome, bacterial growth, and glycoside hydrolase family 32 (GH32) gene abundance, and further examined the association between dietary intake and GH32. Kes and Inu co-supplementation selectively increased *Bifidobacterium adolescentis* and *B. longum*, harboring the GH32 genes *inuA* and *cscA*, respectively. Growth assays revealed that *B. longum,* which expresses *cscA*, grew only on Kes, whereas *B. adolescentis,* which expresses *inuA*, showed growth on Kes and Inu. Only responders—participants showing increases in both species—exhibited consistent upregulation of GH32 genes and were associated with higher retinol and C16:3 (n-6) fatty acid intake, as well as greater green leafy vegetable and canned tuna consumption. This study provides insights into species level responses to prebiotics, supporting personalized dietary strategies for gut microbiota modulation.

## 1. Introduction

The gut microbiota plays an essential role in maintaining metabolic, immune, and neural homeostasis of the host. Dysbiosis, which refers to the disruptions in the composition and function of the microbiota, has been strongly associated with a wide range of diseases, including inflammatory bowel disease (IBD), obesity, type 2 diabetes, autism spectrum disorders, cancer, allergic diseases, and liver disorders [1,2,3]. Owing to its close association with these conditions, increasing attention has been directed toward interventions targeting the gut microbiota. Among various strategies that are aimed at modulating the gut microbiota, dietary intake of prebiotics has gained considerable interest. A prebiotic is defined as “a selectively fermented ingredient that results in specific changes in the composition and/or activity of the gastrointestinal microbiota, thus conferring benefit(s) upon host health” [4]. Prebiotics have been extensively studied as part of dietary interventions aimed at disease prevention and maintenance of health. Recent studies have demonstrated that prebiotics not only improve dysbiosis but also exert beneficial effects on various disease conditions [5].

Fructans represent one of the most well-characterized classes of prebiotics. They consist of linear chains of fructose units linked to a sucrose molecule via β (2→1) glycosidic bonds. Fructans vary in their degree of polymerization (DP), with short-chain fructans known as fructo-oligosaccharides (FOS) and long-chain fructans referred to as inulin (Inu). Short- and long-chain fructans exhibit distinct fermentation characteristics, with different bacterial species responsible for their degradation depending on the DP [6,7].

Among FOS, 1-Kestose (Kes, DP = 3) has been shown to strongly promote the growth of *Bifidobacterium* species, with greater efficacy than other FOS such as Nystose (DP = 4) [8,9]. Intervention studies in humans have indicated that Kes intake modulates gut microbiota and provides physiological benefits, including reductions in fasting insulin levels [10,11]; alleviation of allergic and atopic symptoms [12,13]; and improvements in IBD [14], sarcopenia [15], and pancreatic cancer [16]. Moreover, long-chain fructans such as Inu (DP up to 60) have been reported to improve metabolic syndrome [17], obesity and dyslipidemia [18], and metabolic-associated fatty liver disease [19], as well as enhance calcium absorption [20].

Both short- and long-chain fructans are metabolized by gut bacteria such as *Bifidobacterium* spp. through the action of enzymes belonging to the glycoside hydrolase family 32 (GH32). However, the presence and activity of GH32 genes vary among bacterial species [21]. These species-specific differences may contribute to a complementary and broader activation of the gut microbiota when short- and long-chain fructans are co-administered. Our previous study demonstrated that combined intake of Kes and Inu synergistically enhanced their preventive and therapeutic effects in an allergic mouse model [22,23]. Similarly, a human trial showed that their co-administration promoted calcium absorption and increased bone mineral density [24]. Although several studies have examined the effects of combined administration of short- and long-chain fructans, no human study has yet comprehensively investigated how the gut microbiota responds to their co-administration.

Considerable inter-individual variability has been observed in gut microbiota responses to prebiotics. Recent evidence suggests that this variability may be influenced by host dietary patterns and nutrient intake status [25]. However, studies that simultaneously evaluate interactions between prebiotics and dietary factors in shaping the gut microbiota remain scarce.

Therefore, in the present study, we conducted a double-blind, randomized controlled trial (RCT) in healthy adults to comprehensively assess changes in the gut microbiota following combined intake of Kes and Inu. We focused on bacterial species harboring GH32 family enzymes that are involved in fructan degradation to elucidate their association with individual responsiveness to prebiotics. Additionally, dietary intake was assessed in participants who showed marked shifts in gut microbiota composition, to explore how dietary factors may influence the relationship between prebiotics and gut microbial responses.

## 2. Materials and Methods

### 2.1. Ethical Approval

The study protocol was approved by the Ethics Committee of Nagoya University of Arts and Sciences (approval number: 772). The study was conducted in accordance with the Declaration of Helsinki and the Ethical Guidelines for Medical and Biological Research Involving Human Subjects in Japan. The protocol was registered with the Japan Registry of Clinical Trials (jRCT) (registration number: jRCT1041240180).

### 2.2. Study Design and Participants

Overall, 40 healthy adult volunteers were enrolled in this RCT. Inclusion criteria were as follows: (1) no current diagnosis of any gastrointestinal disorder; (2) no intake of probiotic or prebiotic supplements within the past week; and (3) no antibiotic use within the past week. All participants provided written informed consent after receiving a full explanation of the study objectives and procedures, and their personal information was anonymized to ensure confidentiality.

### 2.3. Randomization and Blinding

Participants who met all inclusion criteria were randomly assigned in a 1:1 ratio to either the prebiotic or placebo group based on random numbers generated using Microsoft Excel, following the method of Oe et al. [26]. Randomization was performed by an independent university staff member not involved in the study. Allocation concealment was maintained using a password-protected file, and both participants and investigators were blinded to group assignments throughout the intervention period.

### 2.4. Intervention and Study Procedure

Participants in the prebiotic group (Prebio group) received a mixture of fructans consisting of 3 g/day of Kes (DP3, 98% purity; Itochu Sugar Co., Ltd., Hekinan, Japan) and 3 g/day of Inu (average DP 8-13; Inulia^®^, Teijin, Japan) for 4 weeks. Participants of the placebo group received 3 g/day of maltose (food-grade; Awajiya, Osaka, Japan) and 3 g/day of cornstarch (food-grade; Hinokunishokuhin, Kumamoto, Japan). All powders were pre-weighed, mixed, and packed in weekly doses in opaque aluminum sachets to maintain blinding. Participants were instructed to consume 6 g/day.

At baseline (week 0), participants completed a dietary assessment using the Brief Self-administered Diet History Questionnaire (BDHQ) [27,28]. Fecal samples were collected at weeks 0 and 4 using FS-0015 collection kits (TechnoSuruga Laboratory Co., Ltd., Shizuoka, Japan). Participants were instructed to maintain their usual dietary and lifestyle habits during the intervention. Adverse events and study withdrawals were monitored throughout the study period.

### 2.5. DNA Extraction and 16S rRNA Gene Sequencing

DNA was extracted from fecal samples using the QIAamp PowerFecal Pro DNA Kit (QIAGEN, Hilden, Germany) according to the manufacturer’s instructions. 16S rRNA gene sequencing was conducted in accordance with previously established methods [29]. Specifically, the V3–V4 region of the bacterial 16S rRNA gene was amplified using primers Pro341F and Pro805R (Supplementary Table S1). PCR amplification was performed using TaKaRa Ex Taq^®^ Hot Start Version (Takara Bio Inc., Shiga, Japan) under the following conditions: initial denaturation at 94 °C for 2 min; 30 cycles of denaturation at 94 °C for 30 s, annealing at 55 °C for 30 s, and extension at 72 °C for 30 s; followed by a final extension at 72 °C for 5 min.

Indexed libraries were then prepared and sequenced on the Illumina MiSeq platform (2 × 300 bp paired-end reads) using the MiSeq Reagent Kit v3. Sequencing was performed by Bioengineering Lab Co., Ltd. (Sagamihara, Japan). The 16S rRNA gene NGS datasets were deposited in the NCBI Sequence Read Archive (SRA) (accession number: PRJNA1301572).

### 2.6. Bioinformatics Analysis

Bioinformatics analysis was conducted according to previously established methods [23]. Specifically, sequence data were analyzed using QIIME2 (version 2022.2) [30]. Quality filtering and denoising were performed using the DADA2 plugin with the following parameters (p-trunc-len-f = 240 and p-trunc-len-r = 240) [31]. Taxonomic classification was performed using the qiime feature-classifier classify-sklearn command with default parameters [32], and taxonomy was assigned based on the Greengenes2 reference database (version 2022.10) [33]. Alpha diversity indices were calculated, and principal coordinate analysis (PCoA) was conducted using weighted UniFrac distances. Beta diversity was evaluated using the qiime diversity beta-group-significance command.

### 2.7. Quantitative Analysis of Fecal Microorganisms Using Quantitative PCR

We aligned the nucleotide sequences of GH32 genes, specifically *inuA* and *cscA*, along with their homologs found in *Bifidobacterium adolescentis* and *B. longum* strains available in the NCBI database. Based on the consensus regions identified from these alignments, primer sets were designed to selectively amplify GH32 gene fragments specific to *B. adolescentis* and *B. longum* (Figures S1 and S2).

Quantitative PCR (qPCR) was performed to determine the copy numbers of GH32 genes specific to *B. longum* and *B. adolescentis*, along with the total bacterial 16S rRNA genes. Amplification was carried out using a QuantStudio 3 system (Thermo Fisher Scientific, Waltham, MA, USA) with the PowerTrack SYBR Green Master Mix (Thermo Fisher Scientific) according to the manufacturer’s instructions.

The qPCR reactions comprised an initial denaturation at 95 °C for 2 min and a final extension at 72 °C for 1 min. Each assay consisted of 40 cycles of 95 °C for 10 s, annealing for 15 s, and 72 °C for 15 s. The annealing temperature was set at 65 °C for *inuA* and at 60 °C for both *cscA* and 16S rRNA. Primer sequences are listed in Supplementary Table S1 [34].

Absolute quantification was achieved using PCR amplicons derived from fecal DNA samples as external standards. The *inuA* and *cscA* levels were defined as the copy number of each gene divided by that of the 16S rRNA gene and multiplied by 10^3^. The change in *inuA* and *cscA* levels was calculated as the difference between post-intervention and baseline values (i.e., post-intervention level–baseline level).

### 2.8. Bacterial Strains and Growth Conditions

The type strains *B. longum* subsp. longum JCM 1217 and *B. adolescentis* JCM 1275 were obtained from the Japan Collection of Microorganisms (JCM). All strains were cultivated using reinforced flora (RF) medium—a modified brain heart infusion (BHI) broth based on a previously described formulation [35]. The RF medium consisted of BHI broth (Thermo Fisher Scientific, Waltham, MA, USA) supplemented with 5 g/L yeast extract (Becton Dickinson, Sunnyvale, CA, USA), 5 g/L K_2_HPO_4_ (Fujifilm-Wako, Osaka, Japan), 8 g/L glucose (Fujifilm-Wako), 0.5 g/L L-cysteine hydrochloride (Sigma Aldrich, St. Louis, MO, USA), 1 g/L Tween 80 (Tokyo Kasei Chemical Co., Tokyo, Japan), 0.005 g/L hemin (Fujifilm-Wako), 0.002 g/L vitamin K_1_ (Fujifilm-Wako), 0.001 g/L resazurin sodium salt (Tokyo Kasei Chemical Co.), 0.025 g/L sodium acetate (Fujifilm-Wako), and 0.01 g/L MgSO_4_·7H_2_O (Fujifilm-Wako), with the pH adjusted to 6.8. As required, 0.25% (*w*/*v*) Kes (<98% purity; Itochu Sugar Co., Ltd., Hekinan, Japan) and 0.25% (*w*/*v*) Inu from chicory (Sigma-Aldrich) solutions, sterilized using a 0.2-µm cellulose acetate filter (DISMIC-25CS, Advantec, Tokyo, Japan), were added to the RF medium before incubation.

For growth assays, each strain was first precultured in RF medium containing 0.25% (*w*/*w*) Kes and 0.25% (*w*/*w*) Inu at 37 °C for 20 h under anaerobic conditions using the AnaeroPack system (Mitsubishi Gas Chemical Company, Tokyo, Japan). The resulting cultures were diluted 10-fold with sterile water (180 µL water + 20 µL culture), and the OD_660_ was measured. Each bacterial suspension was then further diluted in RF medium to achieve a starting OD_660_ of 0.1.

First, 300 µL of RF medium and 80 µL of either 2.5% (*w*/*w*) fructan or 2.5% (*w*/*w*) sucrose was dispensed into each well of a 96-deep well plate (AxyGen Scientific, Union City, CA, USA). Wells were inoculated with 20 µL of the adjusted bacterial suspension (final substrate concentration: 0.5%) and incubated at 37 °C under anaerobic conditions (n = 6). At 27 h post-inoculation, 20 µL of each culture was transferred to 180 µL of sterile water in 96-well flat-bottomed plates (4845-96F; Watson Bio Lab, San Diego, CA, USA), and turbidity was assessed by measuring OD_660_ using a microplate reader (SpectraMax M2; Molecular Devices, San Jose, CA, USA). Growth levels were corrected for non-substrate background by subtracting the OD_660_ values of the no-carbon control (only RF medium). Relative growth was then calculated by normalizing the background-corrected growth in each substrate to that obtained with sucrose, which was set to 100% within the same experiment.

### 2.9. Determination of Short-Chain Fatty Acid (SCFA) and Lactate Concentrations in Fecal Samples

SCFAs (acetate, propionate, and butyrate) and lactate concentrations in fecal samples were determined in accordance with previously established methods [23]. The carboxyl group of SCFAs and lactate in each fecal sample was labeled with 2-nitrophenyl hydrazide using a Short- and Long-Chain Fatty Acid Analysis Kit (YMC, Kyoto, Japan) following the improved kit protocol. Briefly, a 20–50 mg fecal sample was weighed into a 1.5 mL microcentrifuge tube (Eppendorf, Hamburg, Germany). Thereafter, 1 mL of PBS was added to the tube on ice, and the tube was vortexed for 30 s. The tube was centrifuged at 15,000 rpm at 4 °C for 2 min. The supernatant was used for the labeling reaction. Here, 50 µL of the supernatant was mixed with 50 µL of PBS, 200 µL of 2 mM caproic acid (Fujifilm-Wako) as an internal standard, 200 µL of 20 mM 2-nitrophenylhydrazin (in water), and 200 µL of 0.25 M N-(3-dimethylaminopropyl)-N′-ethylcarbodiimide hydrochloride HCl [in ethanol, with an equal volume of 3% pyridine in ethanol (*v*:*v*)]. The mixture was heated at 60 °C for 20 min. Thereafter, 200 µL of 15% (*w*/*v*) potassium hydroxide was added, and the mixture was incubated at 60 °C for 15 min. The reaction mixture was added to 4 mL of a 3.8:0.4 (*v*:*v*) mixture of 0.03 M PBS (pH 6.4):0.5 M hydrochloric acid and then filtered through a 0.45-µm filter (PTFE, Puradisc™ 13 mm Syringe Filters, Whatman, Maidstone, Kent, UK). Subsequently, SCFAs derivatives were extracted using 5 mL of diethyl ether. The diethyl ether layer was evaporated to dryness under a nitrogen stream at room temperature. The residue was dissolved in 200 µL of methanol, and 10 µL of each sample was analyzed using HPLC to quantify the major SCFAs. Chromatographic separations were performed using an HPLC Nexera XL series HPLC Instrument (Shimadzu, Kyoto, Japan) and a YMC-Pack FA HPLC column (6.0 mm I.D. × 250 mm; YMC). The solvent system for elution consisted of 30:16:54 (*v*:*v*:*v*) acetonitrile (Fujifilm-Wako)–methyl alcohol (Fujifilm-Wako)–ultrapure water. The duration of the elution program was 20 min at a flow rate of 1.2 mL/min, and the column temperature was maintained at 50 °C. The eluents were monitored at 400 nm to detect derivatized 2-nitrophenylhydrazine.

### 2.10. Dietary Assessment

Dietary intake data were collected at baseline using the BDHQ, a validated tool designed to estimate habitual food and nutrient intake among Japanese adults over the preceding month. The BDHQ comprises 80 items and enables estimation of intake for 58 food groups and more than 100 nutrients. Standard portion sizes were based on typical Japanese recipes. Intake of dietary supplements was not included in the analysis.

Both nutrient and food group intakes were energy-adjusted to grams per 1000 kcal using the density method [36] prior to statistical analysis.

### 2.11. Statistical Analysis

All statistical analyses were performed using GraphPad Prism version 10.0.6 (GraphPad Software, San Diego, CA, USA). For in vitro growth assays of *Bifidobacterium* strains, data were analyzed using a parametric unpaired *t*-test. All other data were analyzed using non-parametric methods: between-group comparisons were performed using the Mann–Whitney U test, whereas within-group comparisons before and after the intervention were analyzed using the Wilcoxon signed-rank test. Spearman’s rank correlation coefficients were used to evaluate associations between dietary factors and microbiota indices. A *p* of < 0.05 was considered statistically significant, and a *p* of < 0.1 was interpreted as indicating a trend.

## 3. Results

### 3.1. Participant Details and Baseline Characteristics

**Figure 1:**
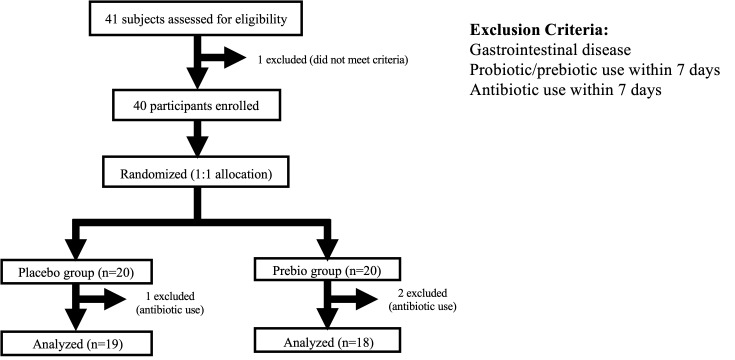
Flow diagram of participant enrollment, randomization, and analysis according to the study protocol.

Figure 1 shows the flow chart of the study. During the study, 2 participants in the Prebio group and 1 in the Placebo group were withdrawn owing to antibiotic use, resulting in 37 participants (18 Prebio, 19 Placebo) included in the final analysis. No participants discontinued the study owing to adverse events such as abdominal pain. All withdrawals were attributed to the use of antibiotics for acute gastrointestinal infections (e.g., viral gastroenteritis), in accordance with the predefined exclusion criteria. Baseline characteristics are summarized in Table 1. All participants were healthy adults, except for one in each group with seasonal allergic rhinitis. There were no significant differences in sex, age, body weight, or BMI between groups. Height was higher in the Prebio group than in the Placebo group, and the total number of supplement intake days was significantly higher in the Placebo group than in the Prebio group.

### 3.2. Baseline and Post-Intervention Fecal Microbiome Diversity and Composition

To evaluate the effects of Kes and Inu supplementation on gut microbiota composition, 16S rRNA gene sequencing was performed on fecal samples collected at baseline and after the 4-week intervention (Post). Changes in both microbial diversity and taxonomic composition were analyzed to determine microbiota responses to prebiotic intake.

**Figure 2:**
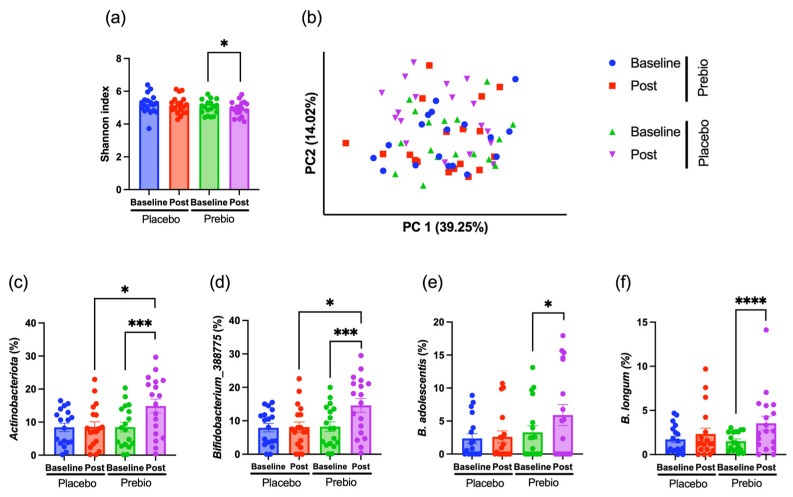
Effects of 1-Kestose and Inulin supplementation on fecal microbiota diversity and composition. (**a**) Shannon index at baseline and post-intervention (Post) in the Prebio and Placebo groups. (**b**) Principal coordinates analysis (PCoA) based on weighted UniFrac distances. The first principal coordinate (PC1) is plotted on the *x*-axis, and the second principal coordinate (PC2) is plotted on the *y*-axis. (**c**) Relative abundance of phylum *Actinobacteria* that were significantly increased in the Prebio group after intervention. (**d**) Relative abundance of genus *Bifidobacterium_388775* within *Actinobacteria* that significantly increased in the Prebio group post-intervention. Relative abundance of species (**e**) *B. adolescentis* and (**f**) *B. longum* within *Bifidobacterium* that significantly increased in the Prebio group post-intervention. *p* < 0.05 (*), *p* < 0.001 (***), *p* < 0.0001 (****). Plots represent individual participants; bars indicate mean ± SEM.

Alpha diversity, measured using the Shannon index, showed a significant decrease post intervention compared with that at baseline only in the Prebio group, whereas no notable change was observed in the Placebo group (Figure 2a). Beta diversity, assessed using PCoA based on weighted UniFrac distances, showed no significant differences between baseline and post-intervention in either group (Figure 2b), suggesting that overall microbiota structure remained relatively stable during the intervention.

Given that fructans such as Kes and Inu selectively promote the growth of certain beneficial bacteria, taxa that increased in response to supplementation were examined. At the phylum level, only Actinobacteria showed a significant increase in the Prebio group post-intervention (Figure 2c). Within Actinobacteria, only the genus *Bifidobacterium_388775* showed a significant post-intervention increase in the Prebio group (Figure 2d). Furthermore, at the species level, only *B. adolescentis* and *B. longum* showed significant increases in relative abundance post-intervention compared with baseline in the Prebio group (Figure 2e,f).

These findings indicate that the observed increases in *Bifidobacterium* taxa were a specific response to prebiotic supplementation, as no such changes were observed in the Placebo group. Despite these taxonomic shifts, fecal SCFAs concentrations at baseline and post-intervention were not significantly different in either group (Supplementary Table S2).

### 3.3. Subgroup Analysis Based on Microbial Response

To explore inter-individual variation in microbial response to prebiotic supplementation, participants in the Prebio group were stratified into two subgroups: responders—defined as individuals who showed increases in both *B. adolescentis* and *B. longum* between baseline and post-intervention—and non-responders—who did not meet this criterion. No significant differences were noted in baseline characteristics—including sex, age, height, body weight, BMI, and the number of supplement intake days—between responders and non-responders (Supplementary Table S3).

**Figure 3:**
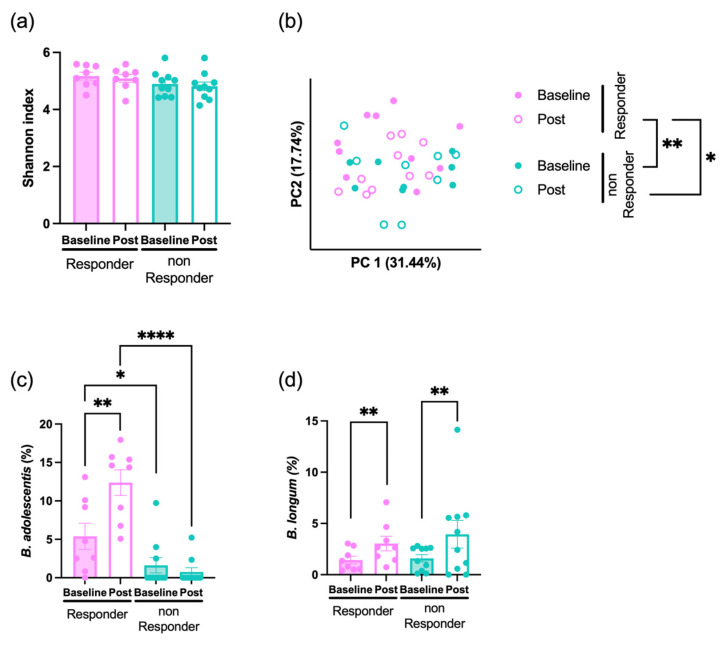
Subgroup analysis based on microbial response to prebiotic supplementation. (**a**) Shannon index at baseline and post-intervention (Post) in responders and non-responders. (**b**) PCoA based on weighted UniFrac distances. The first principal coordinate (PC1) is plotted on the *x*-axis, and the second principal coordinate (PC2) is plotted on the *y*-axis. (**c**,**d**) Relative abundances of (**c**) *B. adolescentis* and (d) *B. longum* at Baseline and Post in responders and non-responders. *p* < 0.05 (*), *p* < 0.01 (**), *p* < 0.0001 (****). Plots represent individual participants; bars indicate mean ± SEM.

Although alpha diversity did not differ between the two subgroups, the beta diversity in post-intervention samples from responders was significantly different from both baseline and post-intervention samples of non-responders (Figure 3a,b), suggesting a distinct compositional shift in the gut microbiota among responders following supplementation.

Next, we examined taxonomic changes in bacterial groups previously identified at the group level. In the responder subgroup, the relative abundance of both *B. adolescentis* and *B. longum* was significantly higher in post-intervention samples than in baseline samples. In the non-responder subgroup, only *B. longum* showed a significant increase post-intervention compared with that at baseline. Moreover, the relative abundance of *B. adolescentis* was significantly higher in responders than in non-responders both at baseline and post-intervention (Figure 3c,d). At baseline, no notable differences were observed between responders and non-responders at the phylum or genus levels, and apart from *B. adolescentis*, only *Blautia_A_141781* unclassified and *Bacteroides_H ovatus* showed significant differences, both of which were more abundant in the non-responder subgroup (Supplementary Figure S3). Thus, only minor compositional differences were observed between the two subgroups.

Despite these responder-specific increases in *Bifidobacterium*, fecal SCFAs concentrations at baseline and post-intervention were not significant in either subgroup (Supplementary Table S4).

### 3.4. GH32 Gene Abundance in Responders and Non-Responders and Fructan Utilization by B. adolescentis and B. longum

To test the hypothesis that the increased abundance of *B. adolescentis* and *B. longum* in responders was associated with the functional capacity of these species to degrade fructans, we examined the abundance of GH32 family genes specific to each taxon.

**Figure 4:**
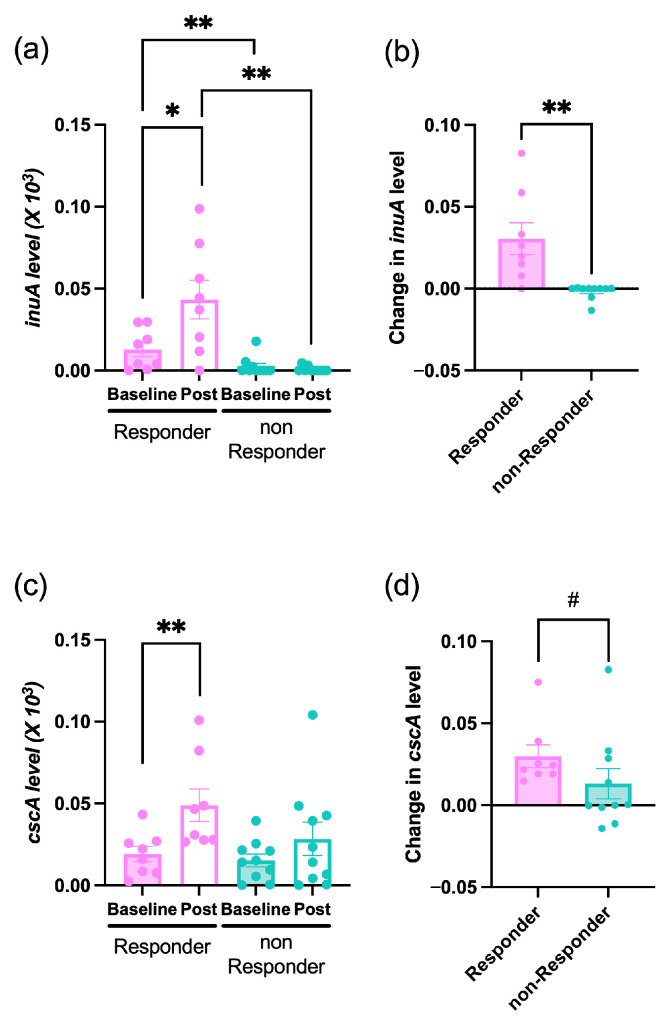
Abundance of GH32 gene homologs in responders and non-responders in the Prebio group. (**a**) *inuA* level at baseline and post-intervention (Post). (**b**) Changes in *inuA* abundance (Δ*inuA*). (**c**) *cscA* level at baseline and Post. (**d**) Changes in *cscA* abundance (Δ*cscA*). Gene abundances were quantified using qPCR and normalized to total bacterial 16S rRNA gene copy number. The change in *inuA* and *cscA* levels was calculated as the difference between post-intervention and baseline values. Bars indicate mean ± SEM. Pink bars, responders; blue bars, non-responders. *p* < 0.05 (*), *p* < 0.01 (**), *p* < 0.1 (#).

The abundance of *inuA* homologs closely mirrored the relative abundance of *B. adolescentis*. It showed a significant increase only in responders and was higher in responders than in non-responders both at baseline and post-intervention (Figure 4a). Moreover, the change in abundance (Δ*inuA*) was significantly greater in responders (Figure 4b). Further, *cscA* homologs also showed a significant increase exclusively in responders, with no significant changes in non-responders (Figure 4c), and the change in abundance (Δ*cscA*) showed a trend toward being greater in responders (Figure 4d).

**Figure 5:**
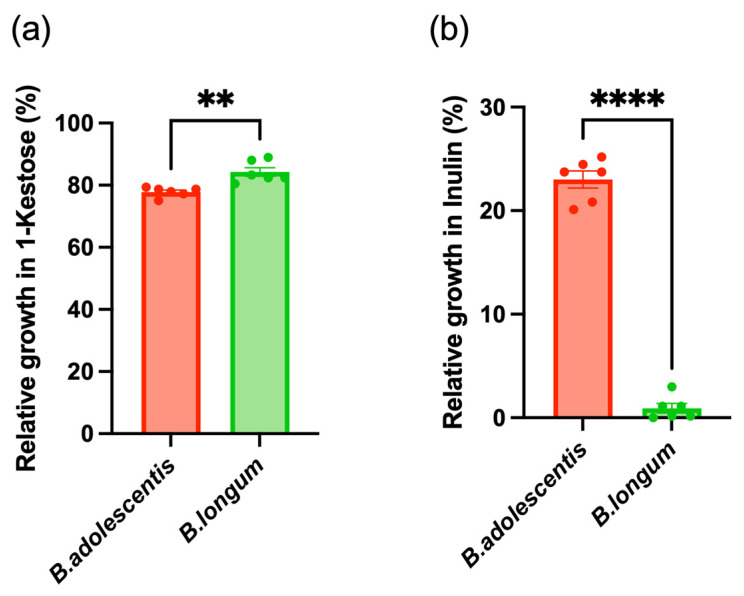
Growth responses of bifidobacteria to fructans. (**a**) Growth of *B. adolescentis* and *B. longum* in Kes-supplemented medium. (**b**) Growth of *B. adolescentis* and *B. longum* in Inu-supplemented medium. Data are presented as mean ± SEM; individual values are shown as dots. *p* < 0.01 (**), *p* < 0.0001 (****).

Following qPCR analysis of *inuA* and *cscA*, we cultured *B. adolescentis* and *B. longum* in media supplemented with Kes or Inu. In Kes-supplemented media, both species exhibited significant growth, although *B. adolescentis* exhibited weaker growth than *B. longum* (Figure 5a). In contrast, in Inu-supplemented media, only *B. adolescentis* showed measurable growth, whereas *B. longum* did not grow (Figure 5b).

### 3.5. Associations Between Nutrient Intake and GH32 Gene Response

**Figure 6:**
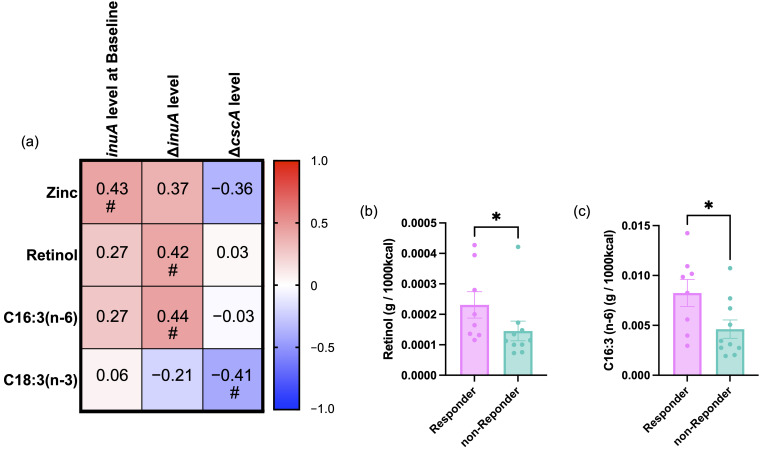
Associations between baseline nutrient intake and microbial GH32 gene response. (**a**) Spearman’s rank correlation between nutrient intake (zinc, retinol, C16:3 (n-6), and C18:3 (n-3)) and GH32 gene abundance markers (*inuA* at Baseline, Δ*inuA*, Δ*cscA*). (**b**) Comparison of baseline retinol intake between responders and non-responders. (**c**) Comparison of baseline C16:3 (n-6) intake between responders and non-responders. *p* < 0.05 (*), *p* < 0.1 (#). Bars indicate mean ± SEM; individual values are shown as dots.

Given that responders exhibited distinct changes in GH32 gene abundance, characterized by higher baseline levels of *inuA* and greater values of Δ*inuA* and Δ*cscA*, we next examined whether these gene markers were associated with baseline nutrient intake assessed using the BDHQ. Spearman’s rank correlation analysis revealed trend-level associations between dietary zinc intake and baseline *inuA* abundance, as well as between retinol and C16:3 ( n-6)) intake and Δ*inuA* levels (Figure 6a). In addition, a negative correlation trend was observed between C18:3 (n-3) intake and Δ*cscA* levels (*p* < 0.1 for all).

To further assess whether these nutrients were linked to microbial responder status, we compared their intake levels between responders and non-responders. Among the candidate nutrients, retinol and C16:3 (n-6) intake were significantly higher in responders than in non-responders (*p* < 0.05), whereas no significant differences were observed for zinc or C18:3 intake (n-3) (Figure 6b,c).

### 3.6. Dietary Food Intake Associated with GH32-Related Microbial Responses

To further characterize dietary factors associated with microbial gene responses, we investigated correlations between the intake of selected food items and three GH32-related markers: Δ*inuA*, retinol, and C16:3 (n-6).

**Figure 7:**
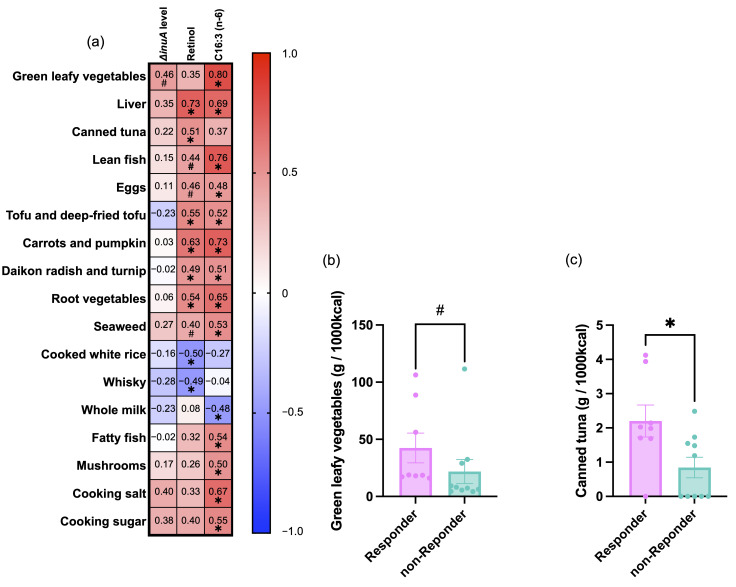
Associations between food group intake and microbial gene response markers. (**a**) Heatmap showing Spearman’s rank correlation coefficients between intake of selected food groups and GH32-related response markers (Δ*inuA*, retinol, and C16:3 (n-6)). (**b**) Intake of green leafy vegetables in responders and non-responders at baseline. (**c**) Intake of canned tuna in responders and non-responders at baseline. Food group intakes were assessed using BDHQ at baseline. *p* < 0.05 (*), *p* < 0.1 (#). Bars indicate mean ± SEM; individual values are shown as dots.

Spearman’s rank correlation analysis revealed trend-level or stronger associations (*p* < 0.1) between these markers and several food items (Figure 7a). Notably, intake of green leafy vegetables was strongly correlated with C16:3 (n-6) and showed a trend-level positive association with Δ*inuA*.

Additional foods showing positive associations with at least one of the three markers included liver, canned tuna, lean fish, eggs, tofu and deep-fried tofu, carrots and pumpkin, daikon radish and turnip, other root vegetables, and seaweed.

In contrast, intake of fatty fish, mushrooms, cooking salt, and cooking sugar was negatively associated with one or more of these GH32-related markers.

Subsequent group comparisons revealed that responders consumed significantly more green leafy vegetables and canned tuna than non-responders (Figure 7b,c), whereas no significant differences were observed for the other food items.

## 4. Discussion

In this randomized, placebo-controlled trial, we evaluated the effects of combined short-chain (Kes) and long-chain (Inu) fructan supplementation on the human gut microbiota. Fructans are generally hydrolyzed by enzymes of the GH32 family, producing fructose monomers that are further metabolized through species-specific pathways. *B. adolescentis* and *B. longum* are known to harbor GH32 genes *inuA* and *cscA*, respectively, which enable them to utilize Inu and Kes, respectively, as carbon sources [37,38]. The intervention selectively promoted the growth of *B. adolescentis* and *B. longum*, along with increases in corresponding GH32 enzymes. These findings suggest that microbial responsiveness to dietary fructans may involve multiple factors, including the presence of relevant catabolic enzymes such as GH32 family fructanases and host dietary patterns.

Both species, which were found to increase in the present study, have been reported to possess immunomodulatory and gut barrier–supporting properties. *B. adolescentis* has been shown to ameliorate dextran sodium sulfate-induced colitis by maintaining tight junction integrity and modulating pro-inflammatory cytokines [39] and may also contribute to antiviral defense against coxsackievirus B3 [40]. *B. longum*, on the contrary, has demonstrated anti-inflammatory effects in IBD models, including the induction of Treg cells and the maintenance of tight junction [41]. Moreover, co-administration of *B. adolescentis* and *B. longum* has been shown to activate a broad range of immune functions, including T-cell responses, NK cell activity, macrophage phagocytosis, and antibody production [42]. These observations suggest that increased levels of these *Bifidobacterium* species may contribute to the maintenance of immune homeostasis and enhancement of host defense mechanisms via the gut microbiota.

Among the metabolites produced by the gut microbiota are SCFAs, including acetate, propionate, and butyrate. These SCFAs act on host immune-related cells—such as dendritic cells, T cells, and macrophages—via G protein–coupled receptors, thereby contributing to immune homeostasis and the regulation of inflammation [43]. *B. adolescentis* and *B. longum* have been reported to produce acetate [44], and their increased abundance is generally considered indicative of enhanced SCFA production in the gut.

In this study, we measured fecal SCFAs concentrations as a non-invasive indicator of the gut environment. However, no significant changes in fecal SCFAs concentrations were observed after the intervention. Although this approach is widely used, the majority of SCFAs produced are absorbed by the intestinal epithelium and distributed systemically [45]. Therefore, fecal concentrations may only partially reflect their actual production. Consequently, potential changes in SCFAs production in this trial might not have been fully captured in fecal measurements. A similar observation was reported in a previous study involving Kes supplementation in children with cow’s milk allergy, where an increase in acetate-producing bacteria did not correspond to changes in fecal SCFAs levels [13]. In addition, as fecal samples were self-collected by participants, minor differences in handling time or storage could also have introduced variability. Future studies incorporating plasma SCFA analysis or standardized collection procedures may provide a more accurate assessment of total SCFA production.

To investigate fructan responsiveness patterns, participants who exhibited increases in both *B. adolescentis* and *B. longum* following the intervention were defined as responders and all others were classified as non-responders. In responders, the abundance of both species, as well as that of their corresponding GH32 genes (*inuA* and cscA), showed a consistent increase. Furthermore, cultivation experiments showed that *B. adolescentis* was able to grow on Inu as well as on Kes, although its growth on Kes was lower than that of *B. longum*. In contrast, *B. longum* showed growth only on Kes. These growth patterns are in line with the observation from previous studies reporting that inulinase of *B. adolescentis* can act on both FOS and Inu, whereas the CscA of *B. longum* exhibits substrate specificity restricted to short-chain fructans [37,38,46]. Collectively, these findings suggest that combined intake of Kes and Inu selectively promotes the growth of *Bifidobacterium* species harboring GH32 enzymes with distinct substrate specificities.

The gut microbiota is strongly influenced by host diet. Therefore, in this study, we investigated nutrients associated with the microbial changes we observed. Responders were characterized by higher baseline levels of *inuA*, together with greater increases in *inuA* and *cscA* during the intervention. Among these features, in particular, we noted associations of retinoic acid (RA) and C16:3 n-6 intake with Δ*inuA*. Among them, retinol is known to regulate T-cell differentiation and antimicrobial peptide expression via its metabolite, RA. Vitamin A deficiency (VAD) is known to severely impair host defense by altering T-cell profiles [47] and reducing the production of antimicrobial peptides [48]. This immunosuppression increases susceptibility to pathogenic infections. In fact, animal models of VAD, including livestock and poultry, have demonstrated reduced resistance to *Escherichia coli* infection [49,50]. Moreover, epidemiological studies in Mexican children have shown that VAD enhances pro-inflammatory cytokine responses during norovirus infection [51]. VAD has also been shown to affect gut microbiota composition. Specifically, VAD models exhibit a decrease in commensal *Bifidobacterium* species and an increase in pathogenic bacteria, such as *Staphylococcus* spp. [52]. These findings suggest that retinoic acid, the bioactive metabolite of retinol, plays a critical role not only in immune regulation but also in maintaining gut microbial homeostasis. Although these observations are based on VAD conditions, they raise the possibility that retinol intake may influence microbial fructan responsiveness through immune and epithelial mechanisms even in healthy individuals.

In contrast to those involving retinol, direct studies on C16:3 n-6 remain limited. However, other n-6 polyunsaturated fatty acids (n-6 PUFAs) are known precursors of pro-inflammatory eicosanoids and have been implicated as risk factors for chronic inflammation when consumed in excess [53]. Nevertheless, appropriate intake of n-6 PUFAs is essential for maintaining immune homeostasis, and their quantitative balance appears to be critical [54]. For instance, n-6 PUFAs have been shown to suppress IL-4-induced increases in intestinal permeability [55] and to protect against the onset of type 1 diabetes in antibiotic-treated mouse models [56]. These findings suggest that retinol and C16:3 n-6 intake may influence gut microbiota composition by modulating host immune and epithelial environments, thereby contributing to inter-individual differences in responsiveness to fructan supplementation.

The food group analysis in this study revealed that the intake of green leafy vegetables and canned tuna was significantly higher among responders. Leafy greens, especially spinach, are rich in C16:3, as reported in the Human Metabolome Database (https://www.hmdb.ca/ accessed on 13 July 2025), and contain other functional components such as β-carotene (a retinol precursor), dietary fiber, and polyphenols. Canned tuna is a good source of n-3 PUFAs (DHA and EPA), vitamin D, and zinc, all of which are known to influence both host physiology and gut microbiota composition [57,58]. In the present study, intake of leafy vegetables was positively correlated with both C16:3 n-6 and Δ*inuA*, suggesting that moderate consumption of leafy greens may enhance fructan metabolism via GH32 enzyme induction.

Although no direct association was observed between canned tuna intake and Δ*inuA*, we cannot exclude the possibility that co-consumed foods or other nutrients present in tuna may contribute to microbial fructan responsiveness. Further investigation is warranted.

This study has several limitations.

First, the observed associations between specific nutrients (e.g., retinol and C16:3 n-6) and microbiome responses are correlational rather than causal. We did not conduct a diet-controlled intervention targeting these nutrients or foods (e.g., green leafy vegetables or canned tuna). Therefore, future studies under strict dietary control, including protocols that vary co-intake of fructans with canned tuna or other nutrient sources, are needed to clarify whether GH32 (*inuA*/*cscA*) responses are differentially modulated. In addition, dietary intake was assessed only at baseline and not after 4 weeks; although most participants reported no major dietary changes during the short intervention, the lack of post-intervention data may have limited the evaluation of diet-related effects. Furthermore, as the dietary assessment was based on self-reported recall and the study did not control for diet, future studies with stricter dietary control are warranted.

Second, this study primarily aimed to investigate the relationship between fructan supplementation, gut microbiota composition, and dietary factors. Accordingly, host physiological parameters were not assessed, and potential links between microbial changes and systemic health outcomes could not be determined. Future studies should therefore include relevant host biomarkers to clarify the physiological significance of microbiome alterations.

Third, because Kes and Inu were administered in combination, it was not possible to determine the specific contribution of each fructan to the observed microbial responses in the human trial. Although the distinct substrate preferences and GH32 enzyme specificities of *B. adolescentis* and *B. longum* were demonstrated in vitro in the present study, their specific roles in humans remain unclear in this study. Therefore, future studies using single-fructan interventions will be necessary to clarify their individual roles in modulating gut microbial dynamics.

Fourth, the intervention lasted only 4 weeks, so the durability of the observed microbial changes remains unknown; longer follow-up is warranted.

Finally, as this was a relatively small-scale trial with a total of 40 participants, the responder and non-responder subgroups were also small (n = 8 and n = 10, respectively), which may limit statistical power and reproducibility. Moreover, the cohort consisted predominantly of young Japanese women (mean age averages, 20 years; women, 94.59%), restricting generalizability across sex, age, and ethnic backgrounds. While BMI was comparable between groups, baseline height differed; although the likelihood of an impact on the outcomes is considered low, a potential influence cannot be ruled out. Consequently, large scale, multicenter studies that include participants with diverse ages, sexes, and health conditions are warranted to validate and extend these findings.

## 5. Conclusions

This randomized, double-blind, controlled trial is the first to examine the effects of combined Kes and Inu supplementation on the human gut microbiota. The results revealed distinct responder and non-responder profiles, with differential expression of GH32 genes—*inuA* and *cscA*—linked to microbial fructan utilization. These enzyme responses were associated with habitual intake of specific nutrients, particularly retinol and C16:3 n-6 fatty acid, and higher consumption of green leafy vegetables and canned tuna. Our findings suggest that both bacterial enzymatic traits and host dietary patterns influence individual responsiveness to prebiotic intake. This study provides foundational evidence for designing personalized dietary strategies to modulate fructan-responsive gut microbes based on microbial function and diet.

## Figures and Tables

**Table 1:** Baseline characteristics of the two groups.

	Placebo Group	Prebio Group	*p*-Value
Sex (Male/Female)	1/18	1/17	>0.9999
Age (years)	20.38 ± 1.12	20.68 ± 0.82	0.4076
Height (cm)	156.30 ± 6.19	160.54 ± 6.47	0.0222
Body weight (kg)	50.27 ± 5.18	52.17 ± 7.93	0.3383
BMI (calculated from BDHQ)	20.57 ± 1.85	20.17 ± 2.18	0.3312
Pre-existing medical conditions	1 participant: hay fever	1 participant: hay fever	>0.9999
Medications and supplements	1 participant: Fexofenadine	1 participant: Fexofenadine 1 participant: Pyridoxal phosphate, Jumihaidokuto	0.6039
Intake Days	27.74 ± 0.13	27.06 ± 0.3	0.0497

Data are presented as mean ± SEM. BDHQ: Brief Self-administered Diet History Questionnaire.

## Data Availability

The original contributions presented in this study are included in the article and Supplementary Materials. Further inquiries can be directed to the corresponding author. The 16S rRNA gene NGS datasets were deposited in The NCBI Sequence Read Archive (SRA) (accession number: PRJNA1301572 (approved on 6 August 2025)).

## Supplementary Materials

The following supporting information can be downloaded at: https://www.mdpi.com/article/10.3390/foods14234090/s1, Supplementary Figure S1: Nucleotide sequence alignment of the GH32 gene (*inuA*) from *Bifidobacterium adolescentis* and related species. Supplementary Figure S2: Nucleotide sequence alignment of the GH32 gene (*cscA*) from *Bifidobacterium longum* and related species. Supplementary Figure S3: Baseline gut microbiota in responder and non-responder subgroups. Supplementary Table S1: PCR primers used in the present study. Supplementary Table S2: Fecal short-chain fatty acid concentrations at baseline and post-intervention in both groups. Supplementary Table S3: Baseline characteristics of the subgroups. Supplementary Table S4: Fecal short-chain fatty acid concentrations at baseline and post-intervention in subgroups.

## Abbreviations

The following abbreviations are used in this manuscript:

BDHQ	Brief Self-administered Diet History Questionnaire
BHI	Brain heart infusion
DP	Degree of polymerization
FOS	Fructo-oligosaccharides
GH32	glycoside hydrolase family 32
IBD	Inflammatory bowel disease
Inu	Inulin
JCM	Japan Collection of Microorganisms
jRCT	Japan Registry of Clinical Trials
Kes	1-Kestose
n-6 PUFAs	n-6 polyunsaturated fatty acids
PCoA	Principal coordinate analysis
qPCR	Quantitative PCR
RA	Retinoic acid
RCT	Randomized controlled trial
RF	Reinforced flora
SCFAs	Short-chain fatty acids
SRA	Sequence Read Archive
VAD	Vitamin A deficiency
