# Supplementary material for: Host Dietary Nutrients Shape GH32-Mediated Microbial Responses to Prebiotic Fructans: A Randomized Trial
Source: Foods. 2025 Nov 28;14(23):4090. doi: 10.3390/foods14234090 (PMC12692294; doi:10.3390/foods14234090)
Supplement: Supplementary file 1 [file foods-14-04090-s001.zip › Supplementary Fig S1.pdf]

```
                            GATTGGCCTGCACAGCCCA
CP047129.1  GAATCCAAGCGACACTGATTGGCCTGCACAGCCCACGCTGGAACAGATCTACAACCGTTTCGGGCGTTCGGGCGCGCATTTCGAACCGTTCGCACGTTGG
CP023005.1  GAATCCAAGCGACACCGATTGGCCTGCACAGCCCACGCTGGAACAGATCTACAACCGTTTCGGGCGTTCGGGCGCGCATTTCGAACCGTTCGCACGTTGG
CP048001.1  GAATCCAAGCGACACCGATTGGCCTGCACAGCCCACGCTGGAACAGATCTACAACCGTTTCGGGCGTTCGGGCGCGCATTTCGAACCGTTCGCACGTTGG
CP156177.1  GAATCCAAGCGACACCGATTGGCCTGCACAGCCCACGCTGGAACAGATCTACAACCGTTTCGGGCGTTCGGGCGCGCATTTCGAACCGTTCGCACGTTGG
AP009256.1  GAATCCAAGCGACACCGATTGGCCTGCACAGCCCACGCTGGAACAGATCTACAACCGTTTCGGGCGTTCGGGCGCGCATTTCGAACCGTTCGCACGTTGG
AP012322.1  GAATCCAAGCGACACCGATTGGCCTGCACAGCCCACGCTGGAACAGATCTACAACCGTTTCGGGCGTTCGGGCGCGCATTTCGAACCGTTCGCACGTTGG
AP012327.1  TAGTACCGGTCGCGATGAGCATTGTGAAATCGTCATCGTCAGCCAGATGCATGTCCGT-----------------GTAATACGCGTTGTTCG--------
CP084012.1  GAATCCGTACGACAATGAATGGGATACGCAGCCGACGCTGGAGCAGATCTATGATCGTTTCGCTCGTCCTGCGGCGCATTTCGAGCCATTCGCTCGCTGG

                                   CCAGTTCCAAGGGCGCTACCA
CP047129.1  ATGAACGATCCGAACGGCCTGTGCCAGTTCCAAGGGCGCTACCACCTGTTCTTCCAGC-TCAACCCGTATGGTTTCGGCTGGGACAATATGCATTGGGGG
CP023005.1  ATGAACGATCCGAACGGCCTGTGCCAGTTCCAAGGGCGCTACCACCTGTTCTTCCAGC-TCAACCCGTATGGTTTCGGCTGGGACAATATGCATTGGGGG
CP048001.1  ATGAACGATCCGAACGGCCTGTGCCAGTTCCAAGGGCGCTACCACCTGTTCTTCCAGC-TCAACCCGTATGGTTTCGGCTGGGACAATATGCATTGGGGG
CP156177.1  ATGAACGATCCGAACGGCCTGTGCCAGTTCCAAGGGCGCTACCACCTGTTCTTCCAGC-TCAACCCGTATGGTTTCGGCTGGGACAATATGCATTGGGGG
AP009256.1  ATGAACGATCCGAACGGCCTGTGCCAGTTCCAAGGGCGCTACCACCTGTTCTTCCAGC-TCAACCCGTATGGTTTCGGCTGGGACAATATGCATTGGGGG
AP012322.1  ATGAACGATCCGAACGGCCTGTGCCAGTTCCAAGGGCGCTACCACCTGTTCTTCCAGC-TCAATCCGTATGGTTTCGGCTGGGACAATATGCATTGGGGG
AP012327.1  --GCACAATAAAGAAGGTCCCGTTTTCGTCTGCATGCGCTTCCAAACGATCGCCTAGCAGCTGCTCGTACACTTCC--------------TTGACAGGAC
CP084012.1  ATGAATGATCCTAACGGCCTGTGTCGTTTCCAAGGACGCTACCATCTGTTCTACCAAT-TCAATCCGTATGGTTTTGGCTGGGACAATATGCACTGGGGC
```

Figure S1. Nucleotide sequence alignment of the GH32 gene (inuA) from Bifidobacterium adolescentis and related species.
The region indicated above the alignment represents the target site selected for primer design. The alignment includes sequences from the following strains and species:
CP047129.1 (*B. adolescentis strain ZJ2*), CP023005.1 (*B. adolescentis strain 6*), CP048001 (*B. longum strain CACC 517*), CP156177.1 *(B. adolescentis strain JCM 19861*), AP009256.1 (*B. adolescentis ATCC 15703*), AP012322.1 (*B. angulatum DSM 20098 = JCM 7096*), AP012327.1 (*B. kashiwanohense JCM 15439 = DSM 21854*), and CP084012.1 (*B. longum strain NBRC 114370*).
