# Supplementary material for: Host Dietary Nutrients Shape GH32-Mediated Microbial Responses to Prebiotic Fructans: A Randomized Trial
Source: Foods. 2025 Nov 28;14(23):4090. doi: 10.3390/foods14234090 (PMC12692294; doi:10.3390/foods14234090)
Supplement: Supplementary file 1 [file foods-14-04090-s001.zip › Supplementary Fig S2.pdf]

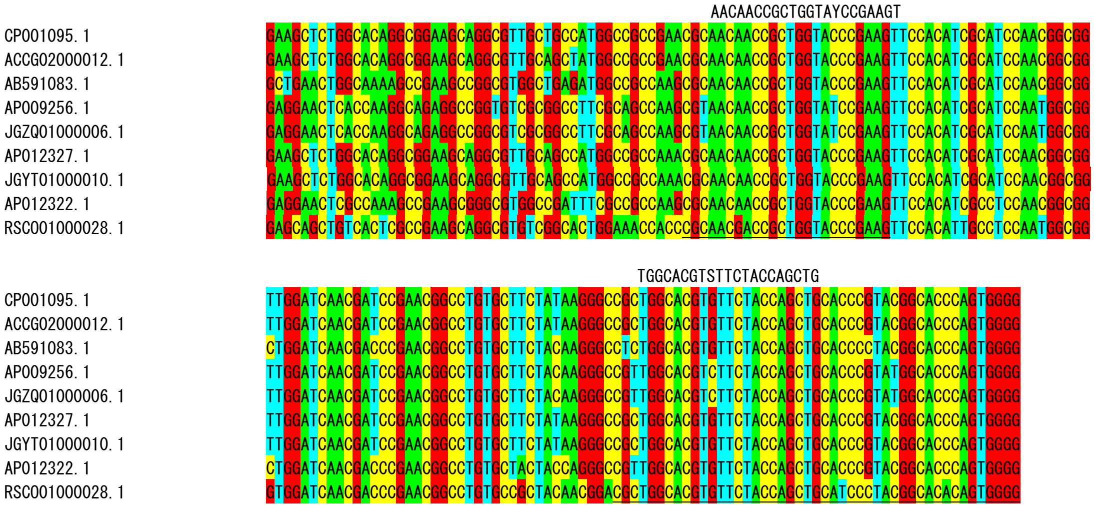

Figure S2. Nucleotide sequence alignment of the GH32 gene (cscA) from Bifidobacterium longum and related species.
The region indicated above the alignment represents the target site selected for primer design. The alignment includes sequences from the following strains and species: The region indicated above the alignment represents the target site selected for primer design. The alignment includes nucleotide sequences from the following strains and species:
CP001095.1 (*B. longum subsp. infantis ATCC 15697*), AP009256.1 (*B. adolescentis ATCC 15703*), AB591083.1 (*B. longum cscA gene for β-fructofuranosidase, strain JCM 1217*), AP012327.1 (*B. kashiwanohense JCM 15439 = DSM 21854*), AP012322.1 (*B. angulatum DSM 20098 = JCM 7096*), JGZQ01000006.1 (*B. stercoris JCM 15918 = DSM 24849*), ACCG02000012.1 (*B. breve DSM 20213 = JCM 1192*), JGYT01000010.1 (*B. catenulatum DSM 16992 = JCM 1194 = LMG 11043*), and RSCO01000028.1 (*B. animalis subsp. lactis strain 2011B*).
