# Supplementary material for: Host Dietary Nutrients Shape GH32-Mediated Microbial Responses to Prebiotic Fructans: A Randomized Trial
Source: Foods. 2025 Nov 28;14(23):4090. doi: 10.3390/foods14234090 (PMC12692294; doi:10.3390/foods14234090)
Supplement: Supplementary file 1 [file foods-14-04090-s001.zip › Supplementary Fig S3.pdf]

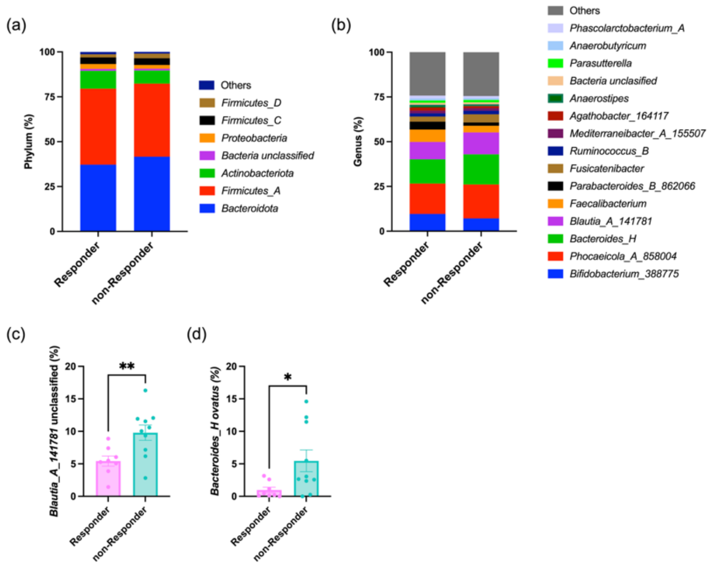

Figure S3. Baseline gut microbiota in responder and non-responder subgroups.

(a) Phylum- and (b) Genus-level compositions at baseline, shown as 100% stacked bar charts. For (a) and (b), taxa with ≥1% relative abundance in both subgroups are displayed; taxa below this threshold are grouped as Others.

Relative abundances of (c) *Blautia_A_141781* unclassified and (d) *Bacteroides_H ovatus* at baseline in the responder and non-responder subgroups. $p < 0.05$ (*), $p < 0.01$ (**). Plots represent individual participants; bars indicate mean ± SEM.
